# The Effects of the 80-hour Workweek on Occupational Hazards

**DOI:** 10.7759/cureus.557

**Published:** 2016-04-03

**Authors:** Doniel Drazin, Lutfi Al-Khouja, Chaim Colen

**Affiliations:** 1 Neurosurgery, Cedars-Sinai Medical Center; 2 Neurosurgery, McLaren Northern Michigan Hospital

**Keywords:** needlestick injury, eyesplash injury, resident training, work-hour restrictions, neurosurgery

## Abstract

**Background:**

The most recent work-hour restrictions were implemented in July 2011 for training physicians. The impact of these regulations on workplace injuries is not yet fully understood. Our goal is to determine the effect of the work-hour limitation on the rates of needlestick and eyesplash injuries.

**Methods:**

Approximately 1200 neurosurgery residents and fellows in the United States were emailed a survey, several times, Sept 2013–February 2014. There were 212 responses across postgraduate years 1–7 and fellowship regarding the rate of needlestick and eyesplash injuries experienced or witnessed before and after July 2011.

**Results:**

Regarding witnessing a needlestick/eyesplash accident: 89.33% of respondents claimed witnessing an injury. Specifically regarding percutaneous injuries (PCIs): before July 2011, 21.77% claimed never witnessing; after July 2011, only 8.9% indicated never witnessing. Specifically regarding eyesplash injuries: comparing the injuries (40.94%) before July 2011 to those (51.94%) after July 2011, the survey indicated an increase in eyesplash injuries.

**Conclusion:**

The results of this survey document that neurosurgery residents/fellows observed (or personally sustained) an increased number of needlestick and eyesplash injuries after implementation of the July 2011 work-hour limitations. Although the last set of reduced-hour regulations have been in place for more than three years, there does not therefore seem to be a safety advantage associated with them regarding a reduction in PCI or eyesplash accidents. This may be due to other confounding factors, not yet affirmatively identified, which warrant additional investigation and identification, directed at preventing future injuries.

## Introduction

The creation and implementation of work-hour limitations in July 2003 for training physicians is a controversial topic that has raised many questions including the sufficiency of current training programs under the new time limitations, the effects on patients being treated by the training physicians (i.e. morbidity and mortality), and the impact on the health and safety of the training physicians themselves (i.e. workplace injury). More work-hour restrictions were put in place in July 2011 limiting hours to a maximum of 16 hours per shift with eight hours off between shifts. This further raised the question of whether these limitations would result in long-term effects during their neurosurgery training such as decreased competence and skill level compared to residents of the same level prior to the placement of the work-hour limitations. Our particular interest revolved around the changes in the rates of injury endured by the training physician, specifically needlestick and eyesplash injuries.

The Occupational Safety and Health Administration (OSHA) estimates that 5.6 million healthcare workers are at risk for exposure to blood-borne pathogens via percutaneous (PCIs), i.e. needlestick and sharp-related injuries; the current estimate of needlestick injuries in the United States ranges from 400,000 to 800,000 per year across all healthcare professionals, with 23% occurring in the operating room [1]. Medical students, interns, and residents account for 7-33% of these injuries every year [2]. The cost to manage a needlestick injury is currently estimated at up to $2,456, depending on the treatment option, highlighting the economic impact of PCIs and the obvious financial benefits from preventing them [3]. A survey of surgical residents performed by Makary et al. showed that 99% of residents experienced at least one needlestick injury before the end of their fifth postgraduate year (PGY) [4]. Ayas et al. identified that PCIs were more frequent in those with extended work hours (OR 1.61, CI 1.46 – 1.78) [3]. It was hoped that the reduction of a resident’s work week to 80 hours would help reduce this high rate of injury. Although some reports have been published addressing the increased risk of injury and exposure with longer working hours, no studies, to our knowledge, have made comparisons between those before and after the 80-hour restrictions were set in place.

The Exposure Prevention and Information Network (EPINet) from the University of Virginia Health System provides an annual report on needlestick and sharp object injuries and blood and body fluid exposures from as early as 1997 [5]. Sharp object injury rates among interns, residents, and fellows began at 9% in 1997, peaked at 18% in 2005, and was 8.9% in the latest report in 2011 [5]. Blood and body fluid exposure rates among interns, residents, and fellows began at 7% in 1997, peaked at 16.1% in 2005, and was 4.6% in the latest (2011) report [1]. The 2011 EPINet report stated that 63.5% of all blood and body fluid exposures occurred through the eyes, while only 7.2% were wearing personal protective equipment (goggles, eyeglasses with side shields, or face shields) [5]. However, there was no breakdown of how many of these occurred in the intern, resident, and fellow groups. Additionally, there was no correlation between the incidence of these exposures compared to the number of hours worked. Investigation of these points can help us evaluate the effect of the work-hour limitations on the safety of physicians in training.

In this study, our objective was to determine the effect of the work-hour limitation on the rates of needlestick and eyesplash injuries sustained by neurosurgery residents and fellows during their training period. Specifically, we looked to observe the change in percutaneous (needlestick) or eyesplash injuries, the nature of these injuries, and how they are managed within the training institution. The reporting protocols for these incidents were also of interest as they help identify methods that encourage injured physicians to report their injuries. The ultimate goal is to provide useful information aimed at developing and implementing prevention strategies to reduce the risk of these workplace injuries. Informed consent was obtained from the participants for this study.

## Materials and methods

All neurosurgery residents and fellows at programs in the United States were emailed a survey several times between September 2013 and February 2014. The survey link was emailed directly by the Congress of Neurological Surgeons (CNS) via an anonymous email blast to neurosurgery residents and fellows on their CNS list. The survey itself was placed on the confidential survey site, Survey Monkey, using the option that instructs Survey Monkey not to record any identifiable information. All responses were voluntary.

The survey was comprised of 16 questions and one comments section regarding the respondent’s demographics, experience with needlestick injury, experience with eyesplash injury, protocol followed after injury, and suggestions for what the protocol should be in handling such injuries. The survey queried residents/fellows regarding their experience with needlestick and eyesplash injuries before and after July 2011.

In our survey, we chose to use July 2011, the date of the most recent work-hour changes, in order to (1) acknowledge that the current residents and fellows were likely not in training in 2003 when the restrictions were first put in place, (2) limit recall bias by surveying residents/fellows about the newer work-hour restrictions instead of asking more senior residents and fellows to compare and remember events from the beginning of their residency, and (3) provide us with current valuable information regarding the impact of the newest work-hour restrictions (which incorporate the previous restrictions placed in 2003).

## Results

A total of 212 neurosurgery residents and fellows from the United States responded to the survey across postgraduate years 1-7 and fellowship. Demographics of the survey respondents are listed in Table 1. Approximately 89.3% claimed they had witnessed a needlestick or eyesplash accident since starting their training. We saw an increase in the number of needlestick or eyesplash injuries reported after July 2011.

**Table 1:** Characteristics of Survey Respondents

Variable	Respondents
Female sex	17.42%
Age	
< 25	1.12%
25-27	13.41%
28-31	43.02%
32-35	27.37%
35-40	11.73%
> 40	3.35%
Postgraduate year	
PGY-1	21.23%
PGY-2	10.06%
PGY-3	12.85%
PGY-4	15.08%
> PGY-5	30.17%
Fellow	7.26%
Practice type	
Academic	98.86%
Private	1.14%
Residency/practice location	
West	22.16%
South	28.41%
Midwest	23.30%
Northeast	26.14%

**Figure 1:**
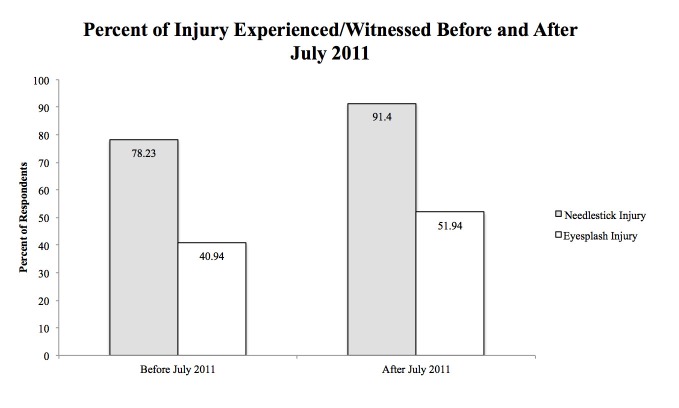
Percentage of Injury Experienced/Witnessed Before and After July 2011 The percent of neurosurgery residents from the survey having either experienced or witnessed a needlestick or eyesplash injury before or after 2011.

Regarding PCIs from needlesticks, 21.8% of physicians claimed they had not witnessed a PCI prior to July 2011. After July 2011, only 8.9% of respondents indicated they have yet to see a PCI. We noted a slight increase in the number of PCIs during emergency procedures: 46.4% to 51.2% (Figure 1; Table 2). Another interesting observation was that after July 2011, 90.5% of physicians said the length of their shift had no impact on the occurrence of PCIs, an increase from 85.3% before 2011. When determining what devices and instruments were most associated with needlestick injuries, suture needle led with a value of 87.6%. The index finger on the non-dominant hand was where most needlestick injuries occurred, with a value of 48.8% (Table 2).

**Table 2:** Survey Results, Needlestick Injuries

Variable	All Subjects (*n* = 212)
Number of residents who incurred/witnessed percutaneous injuries (%)	
Before July 2011	97 (78.23%)
After July 2011	117 (91.40%)
Number of residents who incurred/witnessed percutaneous injuries during an emergency procedure (%)	
Before July 2011	58 (46.40%)
After July 2011	64 (51.2%)
Location of needlestick injury (%)	
Index finger, non-dominant	62 (48.82%)
Index finger, dominant	41 (32.28%)
Other finger, non-dominant	50 (39.37%)
Other finger, dominant	43 (33.86%)
Device or instrument associated with injury (%)	
Suture needle	113 (87.6%)
Scalpel blade	19 (14.73%)
Skin/bone hook	12 (9.30%)
Monopolar	10 (7.75%)
Wire	4 (3.10%)
Scissors	2 (1.55%)
Other	35 (27.13%)

The survey also indicated an increase in the number of eyesplash injuries from 40.9% to 51.9% after the implementation of the newest work-hour restrictions. Moreover, we saw a slight increase in the number of eyesplash injuries during an emergency procedure from 29.1% to 33.3% (Table 3). Approximately 64.2% of physicians reported wearing some sort of protective goggle (or prescription glasses) when they had an eyesplash injury. Additionally, 37.7% of physicians were using a loupe during the eyesplash injury.

**Table 3:** Survey Results, Eyesplash Injuries

Variable	All Subjects (*n* = 212)
Number of residents who incurred/witnessed eyesplash injuries (%)	
Before July 2011	52 (40.94%)
After July 2011	67 (51.94%)
Number of residents who incurred/witnessed eyesplash injuries during an emergency procedure (%)	
Before July 2011	37 (29.13%)
After July 2011	43 (33.33%)
Personal protective equipment (%)	
Prescription glasses	19 (17.92%)
Loupes	40 (37.74%)
Disposable plastic glasses	9 (8.49%)
Eye shield	10 (9.43%)
Other	15 (14.15%)

When asked which treatment or plan they would recommend, 51.7% of respondents believed testing for disease transmission was the best solution for these injuries. Approximately 31.9% never reported the incident even though it had occurred; 48.8% used the immediate and delayed testing as their testing policy after an injury. Additionally, 12.2% of physicians did not know what the institution’s policy was after an injury and never had a discussion about it after the event. Lastly, 7.3% claimed there was no testing required if such an injury occurred. Physicians were also asked if there were any measures taken by the institution in terms of prevention or learning from the event. A large majority (62.9%) said there were not any measures taken by their institution.

The survey addressed the after-incident treatment protocol for a resident who sustained an eyesplash or needlestick injury to prevent transmission of human immunodeficiency virus (HIV), hepatitis B virus (HBV) or hepatitis C virus (HCV) to the injured physician. The patient’s medical history was reviewed and the patient was tested, according to the policy of the specific institution. Survey responders indicated that approximately 56% of patients had no previous medical history of HIV, HBC or HCV. If a patient did in fact test positive for HIV, then 80% of physicians who were offered testing and medication took both. However, if a patient did not test positive for HIV, 14.6% of respondents said they did not proceed with self-testing or medication. It should be noted that the medication regimen lasts for 1-3 months and is a significant burden on the physical health of a resident.

## Discussion

The Ayas et al. study from 2006 reported that extended work hours were associated with an increased rate of PCI but our results showed that this may not be the case [3]. Their survey of 448 interns from July 2002 to May 2003 reported that most respondents cited a lapse of concentration (63.8%) that resulted in the PCI event, while 31% cited fatigue. No similar survey was performed after the implementation of work-hour restrictions in July 2003 or the newest work-hour restrictions in July 2011. Our study, which queried neurosurgery residents and fellows about incidents after the July 2011 reduced work-hour restrictions, showed an increased rate of needlestick injuries, with an increased rate during emergent situations. Similar trends were found with eyesplash injuries with a slight increase during emergency procedures (Figure 1). This information does not mean that longer work hours do not contribute to more injuries. Our findings suggest, however, that although there was a high prevalence of needlestick and eyesplash injuries with longer work hours, the lack of decrease in injury rates after the implementation of work-hour restrictions indicates that other factors are contributing to this. These factors have not yet been affirmatively elucidated in the literature, to our knowledge, especially in regards to neurosurgery interns, residents and fellows. Interestingly enough, this survey shows that a larger number of training neurosurgeons find that the length of their shift does not have an impact on PCI after July 2011. Further investigations on the factors correlated with needlestick and eyeplash injuries would be of great value to the medical education community.

Besides reducing accidental workplace injuries, the work-hour restrictions had a goal of decreasing resident fatigue and errors in patient care. Dumont et al. reported an increased rate of morbidity and mortality on a neurosurgery service after the work-hour limitations [6-7].^ ^In a recent study, Rajaram et al. analyzed data regarding patient outcomes for three years (one year prior to and two years after the work hour restrictions) to determine if the 2011 regulations had improved the mortality or morbidity rates in patients across five surgical specialties (neurosurgery, obstetrics/gynecology, orthopedics, urology, and vascular surgery) [8]. Rajaram et al. reported that according to their multivariable analyses, there were no significant changes in patient mortality or serious morbidity outcome rates after the work hour restrictions were placed [8].^ ^These findings and ours indicate that there is not necessarily a safety advantage in patient outcomes associated with work-hour limitations, as originally thought. 

With the new work-hour restrictions in place, residents across all specialties are limited on the number of hours they can spend to get their cases done. This increased pressure to finish cases within the hour restrictions may be forcing residents to rush their work, increasing the room for error in patient care and rates of accidental self-inflicted injury. Makary et al. concluded that the leading cause of injury was physicians “being in a hurry,” which resulted in accidental self-inflicted injury [4]. Additionally, limiting intern work hours has shifted the workload and created more work for the more senior residents who are not limited to 16 hours per day. Having to juggle additional tasks and spend more time in the operating room may be contributing to the increased injuries identified by the residents and fellows in our survey. Regarding patient care, the work-hour restrictions potentially could be leading to communications errors as patient care is transferred from one intern or resident to another as they rush to comply with the reduced hour limitation.

By limiting work hours, less time can be spent per week training in the operating room and performing procedures, which can result in a relative lack of experience (compared to residents at the same PGY before the implementation of work-hour restrictions). This inexperience may also lead to a greater likelihood of a PCI or eyesplash injury. Dumont et al. commented on the increased reliance on midlevel providers (i.e. nurse practitioners and physicians’ assistants) in the clinical and surgical settings after the work-hour limitations, which have also resulted in reduced exposure and instruction among training physicians [7]. Furthermore, a study by Schwartz and colleagues found that interns had 25.8% less operative cases in 2011-2012 compared to the four years before the placement of the intern work-hour limitations in July 2011 [9]. This inadvertently affected residents by increasing time in the OR, which may also be correlated with the increased rate of injury found in the later years of training as seen in this survey [10].

Over half the physicians surveyed responded that testing was the best plan after a PCI or eyesplash injury. Almost one-third (31.9%) said they have never reported an incident. Perhaps they did not report the injury because they considered a needlestick or eyesplash to be “minor,” felt embarrassed about the incident, became desensitized after enduring multiple injuries, or maybe because they did not have time to place a formal report [6]. If a large number of injuries are not being reported, this raises some concerns including: (1) are injured physicians pursuing testing and/or medication, if indicated, to prevent transmission of HIV, HBC or HCV to themselves, and (2) is there underestimation in reports provided by healthcare institutions. Adding our finding that 62.9% of physicians reported no preventative or learning measures taken by the institution after the injury, it seems that more can be done in this area to help facilitate better education and safety of physicians.

Our focus on improving safety should be maintained and broadened to include identification of any contributing factors and ways to ameliorate them. Another goal should be to encourage reporting of all injuries and to provide the appropriate follow-up. For many residents, a major deterring factor for reporting is the protocol currently in place at their institution. If the protocol is very tedious, time-consuming, and impactful on their day-to-day routine, the residents are probably less likely to report an incident. Having a streamlined protocol for resident reporting, testing, treatment, and closure would encourage participation should an injury occur.

Given the current work hour restrictions, surgery programs have been finding it increasingly difficult to schedule dedicated education time. This is especially the case when it comes to discussing topics such as work-related injury education, prevention, and awareness. Although important, these topics generally take the back seat in the limited time programs have to provide education each week for their residents. Education on work-place injuries can be integrated in and out of the operating room with initiative from senior residents and faculty members. This can be done by ensuring surgeons-in-training are exercising adequate precautions by enforcing use of personal protective equipment, focusing on proper handling of instruments inside and outside the operating room, and encouraging greater proficiency in surgical technical skill. However, this involvement of senior educators in work-place injury prevention does not replace formal lectures and training on the subject. To summarize, a multimodal approach to preventing work-place injury among neurosurgeons in training is needed in the form of formal lectures on injury prevention and safety, involvement of senior educators, and encouraging reporting of injuries in a streamlined fashion.

### Study limitations

Like all survey studies, this study has limitations including response rate, reporting bias, and recall bias. Respondents ranged from PGY1-PGY7 to fellowship with a reasonably balanced response rate for each PGY. While this response distribution may have provided a more objective picture of injuries, treatment, and reporting protocols from different perspectives (based on level of training and experience), PGY1 residents had only to consider their first year of residency while senior residents and fellows had up to eight years of incidents to consider. We tried to eliminate recall bias by asking if they had ever seen an incident and by using July 2011 as the before and after month instead of asking respondents to estimate a number of incidents witnessed. There may have been some recall bias for the more senior residents and fellows, however, due to the “telescoping effect” (wherein dates of incidents become displaced in person’s memory and remote events are remembered as more recent and more recent events are remembered as more remote), because the senior residents/fellows had more years to consider in their responses. Senior residents also have more OR time compared to the more junior residents, which may indicate why there were more injuries in the later years of training. Even given the possibility of recall, the study serves as its own internal control since all respondents likely had some form of recall bias. Additionally, using the changes made in July 2011 as a comparison point rather than July 2003 helped to further decrease the amount of recall bias that may have resulted from this survey.

The questions in the survey were carefully written to prevent overlap (or double counting) between observed and personal exposures. The survey asked about both observed and personal exposures in one question, which provides an estimate of the total number of injuries. Residents are probably more likely to remember almost all of their personal exposures and only some of their observed exposure, which possibly indicates an underreporting of the number of incidents than we found in the survey. If this question were separated into two, observed and personal exposures, then there would have been a concern for double counting.

The survey was kept as short as possible (16 questions) in order to encourage residents and fellows to respond. A longer survey may have provided additional insight but it may have seemed too burdensome and time-consuming, and therefore discouraged some of the residents from responding. A comments section, however, was provided. The interesting recommendations made by residents to improve practices are listed in Table 4. In the future, some of these responses could be rephrased into question form and circulated in a new survey to assess resident response to these suggestions. 

**Table 4:** Interesting Recommendations by Residents to Improve Practices

"There should be a nationwide policy that allows testing of patients without their consent when a needle stick or exposure occurs.”
“Have OR nurses report - they will be the most reliable.”
"The process to be tested and receive medication should be faster, as to not interfere with work and not be another reason not to go to receive treatment.”
"Hastiness of the attending has been the highest cause of needle stick in our institution."
“Currently required to report but [the] process is so arduous (2 hour wait in ED) that most residents and attendings don't want to deal with it. Protocol should be at least mandatory reporting and testing but [the] process needs to take less than 30 minutes to encourage more people to report.”
"It should be made as easy as possible for the resident or staff that was injured.”
”The troubling thing is the exposure source in my state has to consent to viral testing.”
“Hospitals should require the use of protective disposable goggles for the safety of the staff... gloves should also be prick resistant.”

Although this is a preliminary study addressing the correlation between resident work-hour restrictions and injuries, it highlights the need for larger and more in-depth studies characterizing this correlation. In any case, this study addresses the impact of the work-hour restrictions on PCI and eyesplash injuries, the efficacy of reporting protocols currently in place for work-place injuries and focuses on the need to encourage implementation of protocols that promote reporting of such injuries.

### Future outlook

Looking forward, studies identifying the various factors contributing to the increase in needlestick and eyesplash injuries are warranted in order to identify and implement more direct intervention in preventing future injuries. Additionally, institutions are encouraged to provide preventative learning measures (if they are not already doing so or are currently not enforcing them) to identify possible causes of injury and education to prevent them in the future. This in turn can help identify the variables contributing to increasing rates of injury. Further investigation of these findings in neurosurgery, as well as other specialties, is important for the safety of physicians and patients alike.

Nationally, we need to do whatever we can to protect the health and safety of our training physicians. This includes addressing the need for safety shields to prevent eyesplashes (for example, during the placement of extraventricular drains or bolts for neurosurgery residents), an anonymous reporting protocol to get an accurate account of the incident, a standardized protocol for neurosurgery based on rates of transmission, and a residency program protocol for discussion of the incident in a constructive manner.

## Conclusions

Although work-hour limitations have been in place for almost three years, it appears from the present survey that the rates of injury are still increasing. This may be due to some other confounding factors that have not yet been identified. Further attention needs to be given to current reporting protocols at individual institutions. This includes streamlining the protocols so physicians are not hindered from reporting workplace injuries. The end goal is to minimize the incidents, provide care and follow-up for any injuries, and encourage identification and alteration of any modifiable factors.
